# Stick dexterity in carrion crows

**DOI:** 10.3758/s13420-025-00698-9

**Published:** 2025-12-02

**Authors:** Shoko Sugasawa

**Affiliations:** Biosciences Institute, Newcastle University, Henry Wellcome Building, Framlington Place, Newcastle Upon Tyne, NE2 4HH England

**Keywords:** Cognitive ethology, Comparative cognition, Tool use

## Abstract

A recent study trained three tool-naïve carrion crows (*Corvus corone*) to use a stick tool for getting a reward out of a crevice. Automated tracking of tool tips showed gradual changes of their trajectories, demonstrating improved efficiency in their tool dexterity over time (Moll et al. *Current Biology,* 35, 4845-4852, 2025).

Moll et al. (2025) designed a task in which crows were required to “rake out” a pellet reward dispensed at one of three different positions (left, middle, or right) in a horizontal crevice made of transparent Plexiglass. They then tracked the movements of the tip of stick tools using DeepLabCut over the course of up to 2,000 trials. There are a couple of advantages to their methodology: the use of a horizontal and two-dimensional task and the tracking of the tool, rather than the body part of the animals themselves. Unlike popular tool-using tasks used for wild and captive crows (Sugasawa, 2016), the crows in this study used their tool on a horizontal plane. This would have made it easier not only for naturally non-tool-using carrion crows to acquire pellets but also for the authors to keep track of the tool movements, compared with a task where crows extract food from vertical holes. Further, the authors made a sensible choice of tracking the object which animals were manipulating, rather than the animals themselves, as is commonly done in similar image-tracking analyses (Sugasawa, 2016). When applying image tracking to any sort of behaviours in which animals manipulate objects, be it tool use or nest building, inevitably animals often hide behind the object they hold, making continuous tracking of specific body parts (e.g., the crow’s eye or beak) difficult or even infeasible. Thanks to the smart design of the setup and the practical decision to track the tool, the authors successfully tracked and documented that crows’ tool trajectories became less erratic and more streamlined over time.

As noted by the authors, there are some similarities between tool use of their carrion crows and that of habitually tool-using New Caledonian crows (*Corvus moneduloides*). For example, while all three carrion crows learnt to direct their tool towards pellets successfully, they appeared to develop individual patterns to do so. Over the course of several hundred trials, two crows converged on always extracting the pellet on the right-hand side of the crevice, while the other crow matched the initial position of the pellet to the extracted side of the crevice. This side bias, or the laterality, in these carrion crows is reminiscent of that in New Caledonian crows. Individual New Caledonian crows often show strong laterality during tool use (Weir et al., 2004). Data on this developmental process of laterality and its neural underpinnings would help shed light on the plasticity in neural lateralization. Further, the carrion crows adjusted their grip on tools by tossing the tool briefly and regrasping it. While New Caledonian crows also use this method of tool regrasping, notably, carrion crows did not show any use of substrates (e.g., experimental task, floor), the most common method of adjusting the grip on a tool in New Caledonian crows (Sugasawa, 2016). How useful substrates were to the carrion crows would depend on the physical configuration of the setup, although the desk presenting the apparatus seems perfectly fit for the purpose. It would be interesting to see whether this apparent difference in the manipulative diversity is due to a true behavioural difference between carrion crows and New Caledonian crows, or due to a smaller sample size of this study (i.e. with more carrion crows tested, some use substrates). The question moving forward is, what do these similarities (and other differences) between species mean?

This question can only be answered by clarifying what non-tool-using species, in this case carrion crows, represent in the research into the mechanisms underlying tool use. As they are a naturally non-tool-using species, one can be reasonably certain that they can document the learning process of tool use in individual carrion crows (especially in captivity). Obviously, however, the lack of instinctive tool use is not the sole characteristic of carrion crows as a species, and they have their own evolutionary history that have similarities and differences compared with other non-tool-using crows like rooks (*Corvus frugilegus*). For instance, Moll et al. (2025) described that their carrion crows and rooks (Bird & Emery, 2009) used tools differently. This could be due to several reasons. For instance, there were many differences in the tasks (a horizontal crevice in Moll et al., 2025, vs. a vertical tube in Bird & Emery, 2009) and the presentation of tools (i.e., standing in a holder in Moll et al., 2025 vs. lying flat on ground in Bird & Emery, 2009) between these experiments. Further, carrion crows and rooks differ in their beak shape, with carrion crows’ beaks being straighter relative to rooks’ (Matsui et al., 2016). As crows manipulate tools in their beaks, their beak shape affects factors like how securely they can hold a stick in beaks and if/how visible the working end of the tool is during tool deployment (Matsui et al., 2016). There are likely further differences between carrion crows and rooks in their ecology and sociality that may affect their tool-using techniques and performance in the laboratory setting.

These two species, as well as New Caledonian crows and many other Corvus, however, share an important trait: the use of sticks as nest material. Although many of these Corvus species might not be natural tool users, they are certainly stick users. As a functional nest is crucial for successful breeding, many crow species must manipulate sticks adequately to form a solid structure that is more than a ‘pile of sticks’. As both tool use and nest building involve manipulation of sticks, do these behaviours affect each other within a species? For example, do carrion crows with the experience of nest building use a stick more efficiently as a tool than ones without nest-building experience? Such considerations into the natural history of individual species, in addition to the presence and absence of natural tool use, could provide information about important factors affecting tool-using behaviour and its neurological mechanisms in the laboratory.

## Data Availability

Not applicable.
